# Impacted third molar surgery in older patients—Is patient´s age really a risk factor for complications?

**DOI:** 10.1007/s00784-024-05975-x

**Published:** 2024-10-07

**Authors:** Florian Dudde, Manfred Giese, Oliver Schuck, Christina Krüger

**Affiliations:** 1 Department of Oral and Maxillofacial Surgery, Army Hospital Hamburg, Lesserstraße 180, 22049 Hamburg, Germany; 2 Department of Neurology, University Medical Center Hamburg-Eppendorf, Martinistraße 52, 20246 Hamburg, Germany

**Keywords:** Impacted third molars, Complications, Age

## Abstract

**Objectives:**

The aim of this study was to analyze the influence of patients´ age on perioperative complications in impacted third molar surgery and how established risk factors are affected by age.

**Materials and methods:**

The clinical findings, digital panoramic radiographs and perioperative data of 200 patients (554 impacted third molars) that had been subjected to tooth extraction, from July 2023 until July 2024, were analyzed. Perioperative complications (Inferior alveolar nerve (IAN) hypesthesia, oroantral communication (OAC), lingual nerve (LN) hypesthesia, postoperative bleeding, postoperative infection) as well as impaction patterns and risk factors (angulation type, bone coverage, depth- and risk scores) were analyzed by age (cut-off 30 years).

**Results:**

The population was divided into two groups by age (Group A =  ≥ 30 years (*n* = 52) vs. Group B =  < 30 years (*n* = 148)). Upper third molars showed significantly deeper bone coverage, higher depth scores, higher risk scores and different angulation types in patients aged < 30 years. Mandibular third molars showed significantly deeper bone coverage, higher depth scores, higher risk scores according and different angulation types in patients aged ≥ 30 years. However, IAN hypesthesia, LN hypesthesia, postoperative bleeding and postoperative infection did not show any significant differences regarding patients’ age.

**Conclusion:**

The current findings suggest that age (cut-off 30 years) does not statistically correlate with a higher risk for postoperative complications in impacted third molar surgery in contrast to recent publications.

**Clinical relevance:**

In contrast to recent publications, the present study falsified a positive correlation between patients’ age and the occurrence of postoperative complications in impacted third molar surgery. Therefore, other risk factors should be investigated in order to minimize these procedure specific complications.

## Introduction

The surgical removal of third molars is considered a standard procedure in dentistry and oral and maxillofacial surgery [1]. These procedures are usually carried out under outpatient conditions and local anesthesia [1, 2]. Most third molar extractions occur around the age of 20, as by this time most of the jaw development is completed and third molars tend to erupt if they are not impacted [3, 4]. Indications for the removal of third molars include pain, infection and orthodontic reasons [5]. However, in some cases, patients choose not to have surgery for a variety of reasons. Furthermore, asymptomatic impacted and displaced third molars are often not extracted.

If third molars are impacted (i.e. due to eruption problems), the surgical removal of these teeth is usually carried out by oral and maxillofacial surgeons [1]. However, the prevalence of impacted third molars and their impaction patterns vary [4, 6]. The different impaction patterns of the third molars strongly affect the risk for complications in extraction surgery [7, 8]. The general surgical risks in third molar surgery include postoperative bleeding and postoperative infection (i.e. infiltrate, abscess) which may lead to deep space infections in the head and neck region [7, 9]. Prevalences of postoperative bleeding (0.3—1.5%) and postoperative infections (0.17—5.73%) vary among different studies [10–12]. Risk factors for the occurrence of postoperative bleeding include, in addition to anticoagulant medications, extensive extractions and osteotomies [13, 14]. Risk factors for postoperative infections include immunodeficiency, systemic diseases such as diabetes mellitus type II and extensive osteotomies, which may be necessary due to deep impaction patterns and angulation types of third molars [15–17].

A procedure-specific risk in upper third molar extraction is oroantral communication (OAC) especially in deeply impacted upper third molars with marginal distance to the maxillary sinus [1, 15]. This requires the need for plastic reconstruction of the OAC intraoperatively and, postoperatively, strict adherence to a ban on blowing the paranasal sinuses, the use of nasal sprays (i.e. Xylometazoline), and a transient ban on flying and diving [18]. In lower third molars, hypesthesia of the inferior alveolar nerve (IAN) and the lingual nerve (LN) are particularly feared complications [19, 20]. With rates up to 8.4% (IAN hypesthesia) and 2% (LN hypesthesia) these complications should not be neglected when discussing perioperative complications in impacted third molar surgery [19–21]. Studies have already identified several risk factors for the occurrence of the complications mentioned (i.e. angulation type, bone coverage, operation time, oral surgeon (DMD) vs. maxillofacial surgeon (MD, DMD)) [6, 13, 15].

However, whether age as a risk factor affects the complication rates in impacted third molar surgery and to what extent known risk factors (i.e. angulation type, bone coverage) are influenced by age has not been sufficiently investigated yet.

Therefore, this study aims to analyze the influence of patients’ age on the most common complications (OAC, IAN and LN hypesthesia, postoperative bleeding or infection) in impacted third molar surgery and how other specific risk factors (i.e. angulation types, bone coverage) are affected regarding this procedure in patients younger/older than 30 years.

## Materials and methods

### Data collection

This retrospective study examined patients with impacted third molars who were operated in the Department of Oral and Maxillofacial Surgery between July 2023 and July 2024. At least one third molar was extracted from each patient in the study. All patients included in the study had impacted (e.g. completely or partially impacted) third molars with a clear indication for surgical extraction. The extractions were carried out under both outpatient and inpatient conditions. The operations were performed under local anesthesia (i.e. standard IAN nerve block) by experienced oral and/or maxillofacial surgeons (> 2 years of experience, > 400 extractions of impacted third molars) with postoperative use of antibiotics (Amoxicillin 1 g 3x/day or Clindamycin 600 mg 3x/day in case of Penicillin-allergy for a total of 5 days). Modified triangular flap through a marginal approach in upper/lower second molars was used as incision design. Vicryl 4–0 sutures were used as standard sutures for all patients in flap closing. All patients were at least 18 years old and fully capable of consenting to the procedure. Exclusion criteria were erupted third molars, incomplete documentation (patient records), poor-quality panoramic radiographs and patients with medical comorbidities. The baseline characteristics such as sex, age and indication were retrospectively identified for each patient. Furthermore, the preoperative panoramic radiographs were analyzed. Perioperative complications (postoperative bleeding, postoperative infection, IAN hypesthesia, LN hypesthesia) were identified throughout the follow-up (two weeks postoperatively). A total of 200 patients with a total of 554 impacted third molars were included in the present study. The population was divided into two groups by age (Group A =  ≥ 30 years (*n* = 52) vs. Group B =  < 30 years (*n* = 148)).

### Radiographic analysis

The Visage 7 Client (7.1.17) program was used for panoramic radiograph analysis. The analysis and documentation of the panoramic radiographs was carried out by the first author (maxillofacial surgeon).

All impacted third molars were classified according to Winter's classification and classified by the FDI World Dental Federation notation [22]. Based on the analysis of the digital panoramic radiographs, an angle determination was made using two lines (long axis of second and third molar). The impaction of the teeth was classified according to the Pell and Gregory system (A, B, C) [23]. A classification of the relationship to ramus mandibulae of third molars was also performed. This classification was based on the relation of the third molar crown size (distance mesial + distal) and the distance from the end of the external oblique ridge to the most external distal point of the second molar. An analysis of the bone coverage in the upper and lower jaw was performed. This was divided into deep (> 3 mm), medium (1—3 mm), superficial (< 1 mm) and a total lack of bone coverage. The smallest distance from the root tips of the upper third molars to the maxillary sinus was measured. Furthermore, the distance to the maxillary sinus was classified into none, low (< 1 mm), medium (1—3 mm) and far (> 3 mm) distance for upper third molars. Regarding potential nerve damage to the IAN, the smallest distance from the root tip of the lower third molar to the inferior alveolar nerve was measured. In addition, the distance was classified in none, low (< 1 mm), medium (1—2 mm) and far (> 2 mm) to the IAN for lower third molars. Furthermore, a risk analysis for surgical extraction of impacted molars according to the Gordon and Pederson scale was performed [24]. Based on this scoring system the extractions were classified into extractions of low, medium and high difficulty.

### Statistical analysis

Descriptive analysis was used to display patients baseline characteristics. Normally distributed continuous variables are presented as mean ± standard deviation and binary variables are using absolute and relative frequencies. Comparison of continuous variables was performed by Student’s t-test. Chi-square test was used for analysis of binary variables. A *p*-value < 0.05 was considered statistically significant. All statistical analyses were performed using the SPSS version 28.0 statistical package (IBM, Markham, Canada).

## Results

A total of 200 patients with 554 impacted third molars were included in the present study. The population was divided into two groups by age (Group A =  ≥ 30 years (*n* = 52) vs. Group B =  < 30 years (*n* = 148)) (Table 1). 75% of all patients were male (Table 1). No significant differences were found regarding the indications for third molar extraction between the two groups, revealing pain as the most common indication for this procedure (Table 2). Regarding the angulation types according to Winter's classification, there were significant differences between the two groups (Table 3). Patients aged ≥ 30 years were significantly more likely to have mesio- and disto-angulated upper third molars with significantly lower frequencies of horizontal- and vertical-angulation types than patients aged < 30 years (Table 3). Furthermore, patients from group A revealed significantly more horizontal angulated teeth 48 (Table 3). For tooth 38 there were no significant differences regarding the angulation types (Table 3).

**Table 1:** Baseline Characteristics

Variable	Total (*n* = 200)	Age ≥ 30 years (*n* = 52)	Age < 30 years (*n* = 148)	*p*-Value
Age (years)	26.54 ± 6.85	35.85 ± 5.89	23.27 ± 3.17	** < 0.001**
Sex				0.710
Male	150 (75.0)	40 (76.9)	110 (74.3)	
Female	50 (25.0)	12 (23.1)	38 (25.7)	

Data are presented as mean (SD) and/or absolute values (percentage)

**Table 2:** Indication for operation

Variable	Total (*n* = 200)	Age ≥ 30 years (*n* = 52)	Age < 30 years (*n* = 148)	*p*-Value
Indication for operation/complaint				
Pain	138 (69.0)	38 (73.1)	100 (67.6)	0.510
Orthodontics	70 (35.0)	14 (26.9)	56 (37.8)	0.295
Infection	34 (17.0)	10 (19.2)	24 (16.2)	0.619
Prosthetics	10 (5.0)	2 (3.8)	8 (5.4)	0.657
Before Deployment	10 (5.0)	3 (5.8)	7 (4.7)	0.651

Data are presented as absolute values (percentage)

**Table 3:** Distribution of molars by angulation types according to Winter classification [22]

Variable	Total (*n* = 200)	Age ≥ 30 years (*n* = 52)	Age < 30 years (*n* = 148)	*p*-Value
Tooth 18	134 (67.0)	24 (46.3)	110 (74.4)	**0.002**
Mesio-angulated	34 (25.4)	10 (41.7)	24 (21.8)	
Disto-angulated	30 (22.4)	4 (16.7)	26 (23.6)	
Horizontal	2 (1.5)	0 (0)	2 (1.8)	
Vertical	68 (50.7)	10 (41.7)	58 (52.7)	
Tooth 28	126 (63.0)	28 (53.8)	98 (66.2)	**0.004**
Mesio-angulated	32 (25.4)	12 (42.9)	20 (20.4)	
Disto-angulated	22 (17.5)	2 (7.1)	20 (20.4)	
Horizontal	2 (1.6)	2 (7.1)	0 (0)	
Vertical	70 (55.6)	12 (42.9)	58 (59.2)	
Tooth 38	140 (70.0)	32 (61.5)	108 (72.9)	0.340
Mesio-angulated	46 (32.9)	10 (31.2)	36 (33.3)	
Disto-angulated	32 (22.9)	10 (31.2)	22 (20.4)	
Horizontal	46 (32.9)	10 (31.2)	36 (33.3)	
Vertical	16 (11.4)	2 (6.3)	14 (12.9)	
Tooth 48	154 (77.0)	28 (53.8)	126 (85.1)	** < 0.001**
Mesio-angulated	64 (41.6)	12 (42.9)	52 (41.3)	
Disto-angulated	32 (20.8)	2 (7.1)	30 (23.8)	
Horizontal	44 (28.6)	12 (42.9)	32 (25.4)	
Vertical	14 (9.1)	2 (7.1)	12 (9.5)	

Relative frequencies were calculated in relationship to the amount of each tooth (number), Data are presented as absolute values (percentage)

Regarding the Pell and Gregory classification, patients aged < 30 years showed significantly higher frequencies of class C in upper third molars than patients aged ≥ 30 years (Table 4). An inverse observation was made for lower third molars (Table 4). Here, class C on tooth 48 was significantly more common in patients aged ≥ 30 years than in patients aged < 30 years (Table 4). Consecutively, there was a significantly higher bone coverage for lower third molars (deep > 3 mm, medium 1 – 3 mm) in patients aged ≥ 30 years (Table 5). Upper third molars showed a thicker bone coverage in patients aged < 30 years (Table 5). In addition to that, upper third molars showed significantly higher frequencies of marginal distance to the maxillary sinus in patients aged < 30 years (Tooth 18: Group A = 15.4% vs. Group B = 32.43% (*p* = 0.006)) (Tooth 28: Group A = 15.4% vs. Group B = 33.8% (*p* =  < 0.001)) (Table 6). This was also accompanied by significantly smaller distances between the root tip of upper third molars and maxillary sinus in patients aged < 30 years (Table 6).

**Table 4:** Distribution of examined molars by depth according to Pell and Gregory classification [23]

Variable	Total (*n* = 200)	Age ≥ 30 years (*n* = 52)	Age < 30 years (*n* = 148)	*p*-Value
Depth—tooth 18	134 (67.0)	24 (46.2)	110 (74.4)	**0.004**
A	24 (17.9)	4 (16.6)	20 (18.2)	
B	42 (31.3)	10 (41.7)	32 (29.1)	
C	68 (50.8)	10 (41.7)	58 (52.7)	
Depth—tooth 28	126 (63.0)	28 (53.8)	98 (66.2)	**0.002**
A	20 (15.9)	10 (35.7)	10 (10.2)	
B	22 (17.5)	4 (14.3)	18 (18.4)	
C	84 (66.7)	14 (50.0)	70 (71.4)	
Depth—tooth 38	140 (70.0)	32 (61.5)	108 (72.9)	0.282
A	38 (27.1)	6 (18.7)	32 (29.6)	
B	58 (41.4)	12 (37.5)	46 (42.6)	
C	44 (31.4)	14 (43.8)	30 (27.8)	
Depth—tooth 48	154 (77.0)	28 (53.8)	126 (85.1)	** < 0.001**
A	28 (18.2)	2 (7.1)	26 (20.6)	
B	80 (51.9)	10 (35.8)	70 (55.6)	
C	46 (29.9)	16 (57.1)	30 (23.8)	

Relative frequencies were calculated in relationship to the amount of each tooth (number), Data are presented as absolute values (percentage)

**Table 5:** Bone coverage

Variable	Total (*n* = 200)	Age ≥ 30 years (*n* = 52)	Age < 30 years (*n* = 148)	*p*-Value
Bone Coverage—tooth 18	134 (67.0)	24 (46.2)	110 (74.4)	** < 0.001**
None	16 (11.9)	2 (8.3)	14 (12.7)	
Superficial (< 1 mm)	58 (43.3)	16 (66.7)	42 (38.2)	
Medium (1–3 mm)	56 (41.8)	6 (25.0)	50 (45.5)	
Deep (≥ 3 mm)	4 (2.9)	0 (0)	4 (3.6)	
Bone Coverage—tooth 28	126 (63.0)	28 (53.8)	98 (66.2)	0.075
None	14 (11.1)	6 (21.4)	8 (8.2)	
Superficial (< 1 mm)	44 (34.9)	12 (42.9)	32 (32.7)	
Medium (1–3 mm)	60 (47.6)	8 (28.6)	52 (53.1)	
Deep (≥ 3 mm)	8 (6.3)	2 (7.1)	6 (6.1)	
Bone Coverage—tooth 38	140 (70.0)	32 (61.5)	108 (72.9)	**0.001**
None	8 (5.7)	4 (12.5)	4 (3.7)	
Superficial (< 1 mm)	58 (41.4)	4 (12.5)	54 (50.0)	
Medium (1–3 mm)	70 (50.0)	22 (68.7)	48 (44.4)	
Deep (≥ 3 mm)	4 (2.9)	2 (6.3)	2 (1.9)	
Bone Coverage—tooth 48	154 (77.0)	28 (53.8)	126 (85.1)	** < 0.001**
None	12 (7.8)	0 (0)	12 (9.5)	
Superficial (< 1 mm)	58 (37.7)	2 (7.1)	56 (44.4)	
Medium (1–3 mm)	80 (51.9)	24 (85.8)	56 (44.4)	
Deep (≥ 3 mm)	4 (2.6)	2 (7.1)	2 (1.6)	

Relative frequencies were calculated in relationship to the amount of each tooth (number), Data are presented as absolute values (percentage)

**Table 6:** Maxillary third molars

Variable	Total (*n* = 200)	Age ≥ 30 years (*n* = 52)	Age < 30 years (*n* = 148)	*p*-Value
Marginal to maxillary sinus—tooth 18	56 (28.0)	8 (15.4)	48 (32.43)	**0.006**
Mean min. Distance to maxillary sinus ± SD (mm)—tooth 18	1.46 (± 1.31)	1.95 (± 1.48)	1.35 (± 1.26)	**0.041**
Marginal to maxillary sinus—tooth 28	58 (29.0)	8 (15.4)	50 (33.8)	** < 0.001**
Mean min. Distance to maxillary sinus ± SD (mm)—tooth 28	1.28 (± 1.21)	1.99 (± 1.46)	1.06 (± 1.04)	** < 0.001**

Data are presented as mean (SD) and/or absolute values (percentage)

However, since patients aged ≥ 30 years showed significantly deeper impacted lower third molars, tooth 38 consequently revealed significantly higher frequencies of marginal distance between the root tip and IAN (Group A = 84.0% vs. Group B = 63.1% (*p* = 0.007)) (Table 7). The frequencies of marginal distance between tooth 48 and IAN as well as the minimum distance measures did not differ between the two groups (Table 7).

**Table 7:** Mandible third molars

Variable	Total (*n* = 200)	Age ≥ 30 years (*n* = 52)	Age < 30 years (*n* = 148)	*p*-Value
Marginal to inferior alveolar nerve—tooth 38	124 (61.0)	42 (84.0)	82 (63.1)	**0.007**
Mean min. Distance to inferior alveolar nerve ± SD (mm)—tooth 38	0.89 (± 0.90)	0.81 (± 1.05)	0.93 (± 0.87)	0.419
Marginal to inferior alveolar nerve—tooth 48	132 (66.0)	32 (69.6)	100 (70.4)	0.912
Mean min. Distance to inferior alveolar nerve ± SD (mm)—tooth 48	0.90 (± 0.83)	0.92 (± 0.92)	0.89 (± 0.79)	0.848

Data are presented as mean (SD) and/or absolute values (percentage)

In addition to that, lower third molars differed significantly between the two groups regarding the relationship with the ramus mandibulae (Table 8). Patients aged ≥ 30 years showed higher rates of class I and III than patients aged < 30 years (Table 8).

**Table 8:** Distribution of examined molars by relationship to ramus mandibulae

Variable	Total (*n* = 200)	Age ≥ 30 years (*n* = 52)	Age < 30 years (*n* = 148)	*p*-Value
Relationship to Ramus mandibulae—38	140 (70.0)	32 (61.5)	108 (72.9)	**0.031**
I	50 (35.7)	12 (37.5)	38 (35.2)	
II	80 (57.1)	14 (43.8)	66 (61.1)	
III	10 (7.1)	6 (18.8)	4 (3.7)	
Relationship to Ramus mandibulae—48	154 (77.0)	28 (53.8)	126 (85.1)	** < 0.001**
I	58 (37.7)	16 (57.1)	42 (33.3)	
II	84 (54.5)	8 (28.6)	76 (60.3)	
III	12 (7.8)	4 (14.3)	8 (6.4)	

Data are presented as absolute values (percentage)

The Gordon and Pederson scale for predicting difficulty of surgical extraction revealed significant differences between the two groups (Table 9). Patients aged ≥ 30 years showed significantly lower risk scores for tooth 18 (Low risk score: Group A = 58.3% vs. Group B = 49.1% (*p* = 0.003)) and tooth 28 (Low risk score: Group A = 78.6% vs. Group B = 32.7% (*p* =  < 0.001) (Table 9). Lower third molars tended to have significantly higher risk scores in patients aged ≥ 30 years (Tooth 38 High risk score: Group A = 50.0% vs. Group B = 24.1% (*p* = 0.020)) (Tooth 48 High risk score: Group A = 35.7% vs. Group B = 28.6% (*p* =  < 0.001)) (Table 9).

**Table 9:** Risk Scale according to Gordon and Pederson scale for scoring predictive difficulty of surgical extraction [24]

Variable	Total (*n* = 200)	Age ≥ 30 years (*n* = 52)	Age < 30 years (*n* = 148)	*p*-Value
Risk Score—18	134 (67.0)	24 (46.2)	110 (74.4)	**0.003**
Low	68 (50.7)	14 (58.3)	54 (49.1)	
Medium	58 (43.3)	8 (33.3)	50 (45.5)	
High	6 (4.5)	2 (8.3)	4 (3.6)	
Risk Score—28	126 (63.0)	28 (53.8)	98 (66.2)	** < 0.001**
Low	54 (42.9)	22 (78.6)	32 (32.7)	
Medium	58 (46.0)	4 (14.3)	54 (55.1)	
High	14 (11.1)	2 (7.1)	12 (12.2)	
Risk Score—38	140 (70.0)	32 (61.5)	108 (72.9)	**0.020**
Low	20 (14.3)	4 (12.5)	16 (14.8)	
Medium	78 (55.7)	12 (37.5)	66 (61.1)	
High	42 (30.0)	16 (50.0)	26 (24.1)	
Risk Score—48	154 (77.0)	28 (53.8)	126 (85.1)	** < 0.001**
Low	18 (11.7)	4 (14.3)	14 (11.1)	
Medium	90 (58.4)	14 (50.0)	76 (60.3)	
High	46 (29.9)	10 (35.7)	36 (28.6)	

Data are presented as absolute values (percentage)

**Fig. 1:**
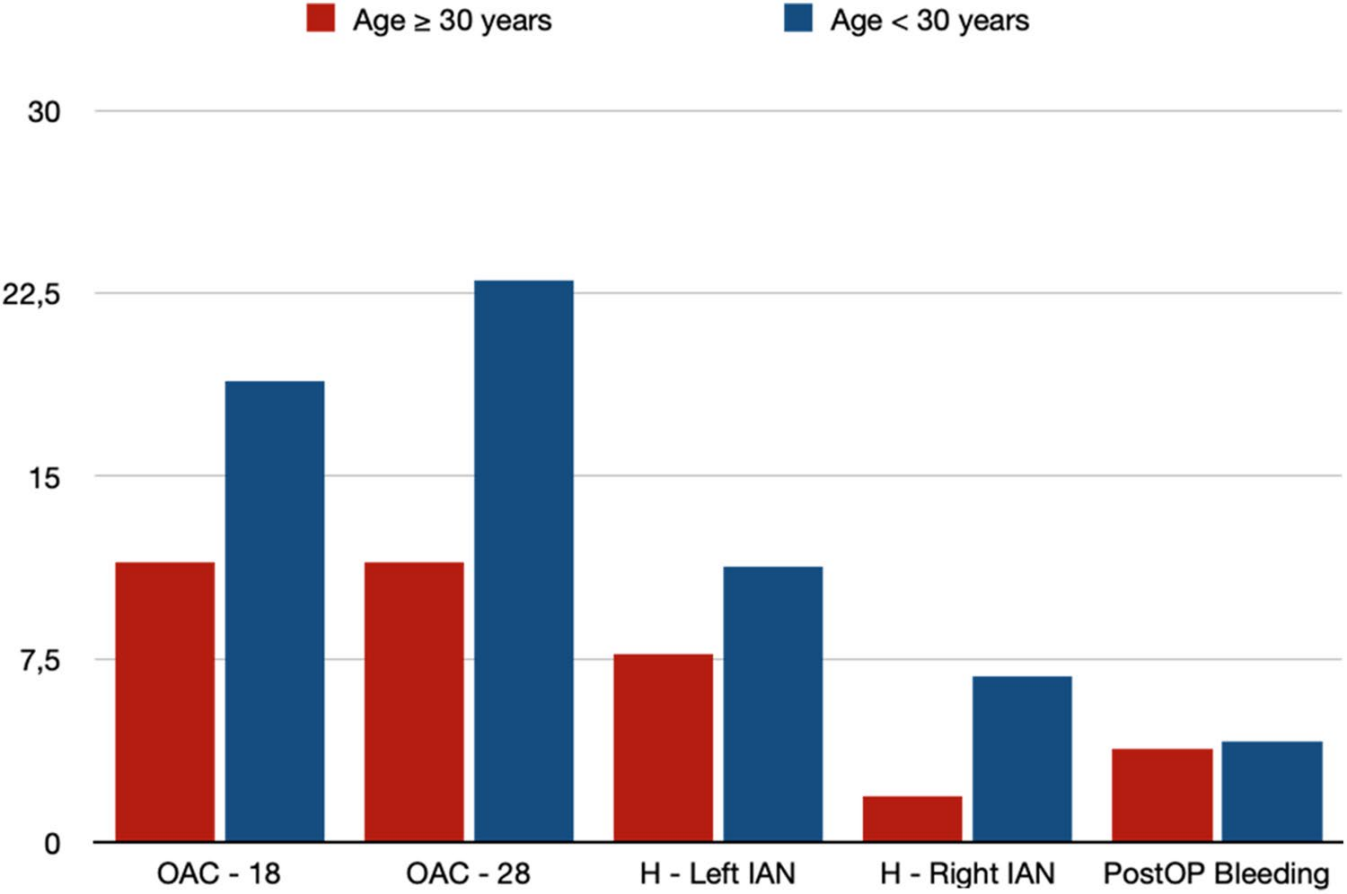
Perioperative complications (Percentage). OAC = Oroantral Communication; H—Left IAN = Hypesthesia left inferior alveolar nerve; H—Right IAN = Hypesthesia right inferior alveolar nerve

Interestingly, there were no significant differences between the two groups regarding perioperative complications (Table 10, Fig. 1). The rates of OAC (Tooth 18: Group A = 11.5% vs. Group B = 18.9% (*p* = 0.223)) (Tooth 28: Group A = 11.5% vs. Group B = 23.0% (*p* = 0.076)) as well as IAN hypesthesia (Left IAN: Group A = 7.7% vs. Group B = 11.3% (*p* = 0.468)) (Right IAN: Group A = 1.9% vs. Group B = 6.8% (*p* = 0.131)) did not show any significant differences. Furthermore, the occurrence of postoperative bleeding (Group A = 3.8% vs. Group B = 4.1% (*p* = 0.843)) and postoperative infection in any region did not differ significantly (Table 10). In addition to that, mean operation time per tooth did not differ between the two groups by age (Group A = 17.14 min. vs. Group B = 18.28 min. (*p* = 0.277)) (Table 10).

**Table 10:** Perioperative complications

Variable	Total (n = 200)	Age ≥ 30 years (n = 52)	Age < 30 years (n = 148)	*p*-Value
Oroantral communication – region 18	34 (17.0)	6 (11.5)	28 (18.9)	0.223
Oroantral communication – region 28	40 (20.0)	6 (11.5)	34 (23.0)	0.076
Hypesthesia IAN left	20 (10.0)	4 (7.7)	16 (11.3)	0.468
Hypesthesia IAN right	11 (5.5)	1 (1.9)	10 (6.8)	0.131
Hypesthesia LN left	0 (0)	0 (0)	0 (0)	/
Hypesthesia LN right	2 (1.0)	0 (0)	2 (1.4)	0.587
Bleeding postoperative	8 (4.0)	2 (3.8)	6 (4.1)	0.843
Infection postoperative—tooth 18	0 (0)	0 (0)	0 (0)	/
Infection postoperative—tooth 28	0 (0)	0 (0)	0 (0)	/
Infection postoperative—tooth 38	5 (2.5)	1 (1.9)	4 (2.7)	0.785
Infection postoperative—tooth 48	2 (1.0)	1 (1.9)	1 (0.7)	0.698
Time per tooth (min)	17.99 (± 6.49)	17.14 (± 6.11)	18.28 (± 6.61)	0.277

Data are presented as mean (SD) and/or absolute values (percentage)

## Discussion

The removal of impacted third molars is a comparatively low-risk procedure in oral and maxillofacial surgery. However, the complication rates (i.e. OAC, transient or permanent hypesthesia IAN/ LN, postoperative infection, postoperative bleeding) may be as high as 30%, depending on the study [7]. The present study included a total of 200 patients who had at least one impacted third molar surgically extracted. A total of 554 impacted third molars were removed. The aim of the present study was to investigate the influence of age (cut-off 30 years) on the perioperative complications after impacted third molar surgery as well as on the established risk factors.

In the present study, there were no significant differences between the two cohorts with regard to OAC. Interestingly, patients aged < 30 years had a higher percentage of OAC for both upper third molars than patients aged ≥ 30 years (non-significant). At the same time, patients aged < 30 years also showed significantly higher bone coverages, higher depths (class C) and lower minimum distances to maxillary sinus regarding upper third molars, so that the increased prevalence of OAC is only consequent. In the course of this, the results of the present study can only confirm the conclusion of Rothamel et al. who suspected a higher degree of impaction with a greater likelihood for OAC [25]. In comparison, the rates of OAC of the present study are slightly below the rates of Rothamel´s study with a rate of up to 24% of OAC after extraction of deeply impacted upper third molars [25].

Focusing on IAN injuries, Sarikov et al. found rates of IAN hypesthesia up to 8.4% following lower third molar extraction [19]. According to their review, age > 24 years is considered a risk factor for this type of complication [19]. In the present study, no significant differences regarding patients' age (cut-off 30 years) were found. In addition to that, patients aged < 30 years showed even higher frequencies in hypesthesia of the IAN. However, all of these cases of hypesthesia showed a decline over time. Permanent hypesthesia was not found in the present study and is considered a rare complication according to Sarikov et al. [19]. Furthermore, Sarikov et al. demonstrated that the risk of IAN hypesthesia is associated with horizontal impaction patterns [19]. However, in the present study, patients aged ≥ 30 years showed higher overall numbers of horizontally impacted lower third molars than patients aged < 30 years. Consequently, the results of the present study contradict the findings of Sarikov et al. regarding age and horizontal impaction as a risk factor for IAN hypesthesia. Another well-established risk factor for the development of IAN hypesthesia is the depth of third molars according to Pell and Gregory classification [11, 15]. In the present study, patients aged ≥ 30 years showed significantly higher rates of Class C than patients aged < 30 years. Consecutively, patients aged ≥ 30 years showed significantly higher bone coverage (deep and medium) and higher rates of marginal distance to IAN in lower third molars. Nevertheless, patients aged ≥ 30 years did not show significantly higher rates of IAN hypesthesia, so that the influence of patients’ age on this procedure-specific complication must be evaluated critically. In their prospective multicenter study, Yamada et al. showed that patients’ age (≥ 32 years vs. < 32 years; OR = 1.428) and impaction depth according to Pell and Gregory (class C vs class A, OR = 3.7622) are significant independent risk factors for postoperative complications after lower third molar extraction [11]. In the present study, there were significant differences regarding the impaction patterns against the background of patients’ age, but without effects on the rates of postoperative IAN hypesthesia.

With regard to the rates of postoperative LN hypesthesia, there were also no differences based on patients’ age in the present study. Only 1% of all patients had transient LN hypesthesia postoperatively. Thus, this rate is overall below the rates of comparable studies with up to 2.2% [26, 27]. In their study, Rieder et al. were also unable to demonstrate a significant correlation between patients’ age and the occurrence of postoperative neurosensory deficits (IAN/ LN hypesthesia), meaning that impaction patterns, angulation types or the surgical experience of the surgeon could possibly play a more crucial role regarding this type of complication [15, 19, 27].

There were also no significant differences between the two groups regarding the rates of postoperative bleeding. A direct comparison shows similar postoperative bleeding rates with other studies [13, 28]. Although age is not significantly associated with increased postoperative bleeding rates in the present study, older people are more likely to take anticoagulant medications [29]. Consequently, the use of anticoagulant medication increases the postoperative bleeding risk, particularly in dentoalveolar procedures such as extraction of impacted third molars with extensive osteotomies [30].

Postoperative infection following third molar extraction is one of the most common complications from this procedure [11, 12]. In the present study, no infections occurred in the upper third molars. These findings are in accordance with the results of the study by Sukegawa et al. [16]. Furthermore, the rates of postoperative infections in lower third molars are comparable to other studies [16, 31]. However, the current findings did not show any differences of postoperative infections by patients’ age. Known risk factors for postoperative infections after third molar surgery are the complexity of the extraction as well as the depth of impaction [16, 31]. Even though patients aged ≥ 30 years showed deeper impaction patterns in lower third molars and consecutively higher risk scores according to Gordon and Pederson difficulty scale for third molar extraction, these findings could not be confirmed regarding the rates of postoperative infections. These findings are in accordance with the results of Sukegawa et al., who was also not able to identify age as a risk factor for postoperative infections following third molar extraction [16]. The findings of the present study are limited by several facts. This study is retrospective in nature and represents a monocentric data analysis. Even though the study is comparable in size to other studies focusing on perioperative complications in impacted third molar surgery, the study population should be considered midsize. Therefore, larger study collectives in a multicenter setting are required in order to confirm these results.

## Conclusion

The aim of this study was to analyze the influence of age on the complications in impacted third molar surgery and how established risk factors are affected by age.

Regarding the cut-off of 30 years, upper third molars showed significantly deeper bone coverage, higher depth scores according to Pell and Gregory, higher risk scores according to Gordon and Pederson and different angulation types in patients aged < 30 years. However, OAC, postoperative infection and postoperative bleedings did not differ. Lower third molars showed significantly deeper bone coverage, higher depth scores according to Pell and Gregory, higher risk scores according to Gordon and Pederson and different angulation types in patients aged ≥ 30 years. However, IAN hypesthesia, LN hypesthesia, postoperative bleeding and postoperative infection did not show any significant differences regarding patients’ age. Therefore, the current findings suggest that age (cut-off 30 years) does not statistically correlate with a higher risk for perioperative complications in contrast to other studies addressing impacted third molar surgery. Consecutively, other risk factors should be examined in more detail in order to minimize these procedure specific complications.

## Data Availability

No datasets were generated or analysed during the current study.

## References

[1] Sifuentes-Cervantes JS, Carrillo-Morales F, Castro-Núñez J, Cunningham LL, Van Sickels JE (2021) Third molar surgery: Past, present, and the future. Oral Surg Oral Med Oral Pathol Oral Radiol 132(5):523–531. 10.1016/j.oooo.2021.03.004 34030996 10.1016/j.oooo.2021.03.004

[2] Ku JK, Kim JY, Jun MK, Jeong YK, Huh JK (2021) Influence of General and Local Anesthesia on Postoperative Pain after Impacted Third Molar Surgery. J Clin Med 10(12):2674. 10.3390/jcm10122674 34204470 10.3390/jcm10122674 PMC8234107

[3] Tuteja M, Bahirwani S, Balaji P (2012) An evaluation of third molar eruption for assessment of chronologic age: A panoramic study. J Forensic Dent Sci 4(1):13–18 23087576 10.4103/0975-1475.99154 PMC3470411

[4] Muhsin H, Brizuela M (2024) Oral Surgery, Extraction of Mandibular Third Molars. 2023 Mar 19. In: StatPearls [Internet]. Treasure Island (FL): StatPearls Publishing 36508551

[5] Marciani RD (2007) Third molar removal: an overview of indications, imaging, evaluation, and assessment of risk. Oral Maxillofac Surg Clin North Am 19(1):1–13. 10.1016/j.coms.2006.11.007 10.1016/j.coms.2006.11.007 18088860

[6] Hashemipour MA, Tahmasbi-Arashlow M, Fahimi-Hanzaei F (2013) Incidence of impacted mandibular and maxillary third molars: a radiographic study in a Southeast Iran population. Med Oral Patol Oral Cir Bucal 18(1):e140–e145 23229243 10.4317/medoral.18028 PMC3548634

[7] Rizqiawan A, Lesmaya YD, Rasyida AZ, Amir MS, Ono S, Kamadjaja DB (2022) Postoperative Complications of Impacted Mandibular Third Molar Extraction Related to Patient’s Age and Surgical Difficulty Level: A Cross-Sectional Retrospective Study. Int J Dent 2022:7239339. 10.1155/2022/7239339 35027927 10.1155/2022/7239339 PMC8749374

[8] Bouloux GF, Steed MB, Perciaccante VJ (2007) Complications of third molar surgery. Oral Maxillofac Surg Clin North Am 19(1):117–28, vii. 10.1016/j.coms.2006.11.013 10.1016/j.coms.2006.11.013 18088870

[9] Yilmaz S, Bas B, Ozden B, Selcuk U, Cengel Kurnaz S (2015) Deep neck infection after third molar extraction. J Istanb Univ Fac Dent 49(2):41–45. 10.17096/jiufd.82633 10.17096/jiufd.82633 PMC5573484 28955535

[10] Candotto V, Oberti L, Gabrione F, Scarano A, Rossi D, Romano M (2019) Complication in third molar extractions. J Biol Regul Homeost Agents 33(3 Suppl. 1):169–172 (DENTAL SUPPLEMENT) 31538464

[11] Yamada SI, Hasegawa T, Yoshimura N, Hakoyama Y, Nitta T, Hirahara N, Miyamoto H, Yoshimura H, Ueda N, Yamamura Y, Okuyama H, Takizawa A, Nakanishi Y, Iwata E, Akita D, Itoh R, Kubo K, Kondo S, Hata H, Koyama Y, Miyamoto Y, Nakahara H, Akashi M, Kirita T, Shibuya Y, Umeda M, Kurita H (2022) Prevalence of and risk factors for postoperative complications after lower third molar extraction: A multicenter prospective observational study in Japan. Medicine (Baltimore) 101(32):e29989. 10.1097/MD.0000000000029989 35960058 10.1097/MD.0000000000029989 PMC9371489

[12] Mukainaka Y, Sukegawa S, Kawai H, Nishida T, Miyake M, Nagatsuka H (2024) Risk factors for post-extraction infection of mandibular third molar: A retrospective clinical study. J Stomatol Oral Maxillofac Surg 101841. 10.1016/j.jormas.2024.101841 10.1016/j.jormas.2024.101841 38521244

[13] AlSheef M, Gray J, AlShammari A (2021) Risk of postoperative bleeding following dental extractions in patients on antithrombotic treatment. Saudi Dent J 33(7):511–517. 10.1016/j.sdentj.2020.09.005 34803294 10.1016/j.sdentj.2020.09.005 PMC8589605

[14] Buchbender M, Schlee N, Kesting MR, Grimm J, Fehlhofer J, Rau A (2021) A prospective comparative study to assess the risk of postoperative bleeding after dental surgery while on medication with direct oral anticoagulants, antiplatelet agents, or vitamin K antagonists. BMC Oral Health 21(1):504. 10.1186/s12903-021-01868-7 34620135 10.1186/s12903-021-01868-7 PMC8499467

[15] Dudde F, Barbarewicz F, Henkel KO (2024) Risk factor analysis for perioperative complications in impacted third molar surgery - a single center experience. Oral Maxillofac Surg. 10.1007/s10006-024-01232-3 38427098 10.1007/s10006-024-01232-3

[16] Sukegawa S, Yokota K, Kanno T, Manabe Y, Sukegawa-Takahashi Y, Masui M, Furuki Y (2019) What are the risk factors for postoperative infections of third molar extraction surgery: A retrospective clinical study? Med Oral Patol Oral Cir Bucal 24(1):e123–e129. 10.4317/medoral.22556 30573720 10.4317/medoral.22556 PMC6344007

[17] Mesgarzadeh AH, Ghavimi MA, Gok G, Zarghami A (2012) Infratemporal space infection following maxillary third molar extraction in an uncontrolled diabetic patient. J Dent Res Dent Clin Dent Prospects. Summer;6(3):113–5. 10.5681/joddd.2012.024 10.5681/joddd.2012.024 PMC3442426 22991649

[18] Azzouzi A, Hallab L, Chbicheb S (2022) Diagnosis and Management of oro-antral fistula: Case series and review. Int J Surg Case Rep 107436. 10.1016/j.ijscr.2022.107436 10.1016/j.ijscr.2022.107436 PMC9403197 35917603

[19] Sarikov R, Juodzbalys G (2014) Inferior alveolar nerve injury after mandibular third molar extraction: a literature review. J Oral Maxillofac Res 5(4):e1. 10.5037/jomr.2014.5401 25635208 10.5037/jomr.2014.5401 PMC4306319

[20] Tojyo I, Nakanishi T, Shintani Y, Okamoto K, Hiraishi Y, Fujita S (2019) Risk of lingual nerve injuries in removal of mandibular third molars: a retrospective case-control study. Maxillofac Plast Reconstr Surg 41(1):40. 10.1186/s40902-019-0222-4 31555619 10.1186/s40902-019-0222-4 PMC6733934

[21] Pippi R, Spota A, Santoro M (2017) Prevention of lingual nerve injury in third molar surgery: literature review. J Oral Maxillofac Surg 75:890–900. 10.1016/j.joms.2016.12.040 28142010 10.1016/j.joms.2016.12.040

[22] Winter GB (1926) Impacted Mandibular Third Molars*.* American Medical Book Co.; St. Louis

[23] Pell GJ, Gregory GT (1933) Impacted mandibular third molars: Classification and modified technique for removal. Dent Dig 39:330–338

[24] Pederson GW. Oral surgery. Philadelphia: WB Saunders, 1988. (Cited in: Koerner KR (1994) The removal of impacted third molars—principles and procedures. Dent Clin North Am;38:255–278. 8206177

[25] Rothamel D, Wahl G, d’Hoedt B, Nentwig GH, Schwarz F, Becker J (2007) Incidence and predictive factors for perforation of the maxillary antrum in operations to remove upper wisdom teeth: prospective multicentre study. Br J Oral Maxillofac Surg 45(5):387–391. 10.1016/j.bjoms.2006.10.013 17161510 10.1016/j.bjoms.2006.10.013

[26] Daware SN, Balakrishna R, Deogade SC, Ingole YS, Patil SM, Naitam DM (2021) Assessment of postoperative discomfort and nerve injuries after surgical removal of mandibular third molar: A prospective study. J Family Med Prim Care 10(4):1712–1717. 10.4103/jfmpc.jfmpc_280_19 34123917 10.4103/jfmpc.jfmpc_280_19 PMC8144789

[27] Rieder M, Remschmidt B, Schrempf V, Schwaiger M, Jakse N, Kirnbauer B (2023) Neurosensory Deficits of the Mandibular Nerve Following Extraction of Impacted Lower Third Molars-A Retrospective Study. J Clin Med 12(24):7661. 10.3390/jcm12247661 38137730 10.3390/jcm12247661 PMC10743649

[28] Rademacher WMH, Rooijers W, Broekman MW, van der Slik BJ, van Diermen DE, Rozema FR. Nabloedingsrisico na verwijdering derde molaar bij gezonde patiënten; een prospectief observationeel klinisch onderzoek (2024) Postoperative risk of bleeding after removal of third molars in healthy patients - a prospective observational clinical study. Ned Tijdschr Tandheelkd 131(7–08):307–315. Dutch. 10.5177/ntvt.2024.07/08.24028 10.5177/ntvt.2024.07/08.24028 38973659

[29] Boccatonda A, Frisone A, Lorusso F, Bugea C, Di Carmine M, Schiavone C, Cocco G, D’Ardes D, Scarano A, Guagnano MT (2023) Perioperative Management of Antithrombotic Therapy in Patients Who Undergo Dental Procedures: A Systematic Review of the Literature and Network Meta-Analysis. Int J Environ Res Public Health 20(7):5293. 10.3390/ijerph20075293 37047909 10.3390/ijerph20075293 PMC10093975

[30] Huang J, Liu J, Shi H, Wu J, Liu J, Pan J (2022) Risk factors for bleeding after dental extractions in patients receiving antithrombotic drugs - A case control study. J Dent Sci 17(2):780–786. 10.1016/j.jds.2021.10.005 35756819 10.1016/j.jds.2021.10.005 PMC9201513

[31] Yue Yi EK, Siew Ying AL, Mohan M, Menon RK (2021) Prevalence of Postoperative Infection after Tooth Extraction: A Retrospective Study. Int J Dent 2021:6664311. 10.1155/2021/6664311 34211554 10.1155/2021/6664311 PMC8208874

